# Possible involvement of IGF‐1 signaling on compensatory growth of the infraspinatus muscle induced by the supraspinatus tendon detachment of rat shoulder

**DOI:** 10.1002/phy2.197

**Published:** 2014-01-13

**Authors:** Tsuyoshi Ichinose, Ronny Lesmana, Atsushi Yamamoto, Tsutomu Kobayashi, Hitoshi Shitara, Daisuke Shimoyama, Yusuke Takatsuru, Toshiharu Iwasaki, Noriaki Shimokawa, Kenji Takagishi, Noriyuki Koibuchi

**Affiliations:** 1 Department of Integrative Physiology, Gunma University Graduate School of Medicine, Maebashi, Gunma, Japan; 2 Department of Orthopaedic Surgery, Gunma University Graduate School of Medicine, Maebashi, Gunma, Japan; 3 Department of Physiology, Universitas Padjadjaran, Bandung, Indonesia

**Keywords:** Animal study, functional compensation, rotator cuff tear

## Abstract

A rotator cuff tear (RCT) is a common musculoskeletal disorder among elderly people. RCT is often treated conservatively for functional compensation by the remaining muscles. However, the mode of such compensation after RCT has not yet been fully understood. Here, we used the RCT rat model to investigate the compensatory process in the remaining muscles. The involvement of insulin‐like growth factor 1 (IGF‐1)/Akt signaling which potentially contributes to the muscle growth was also examined. The RCT made by transecting the supraspinatus (SSP) tendon resulted in atrophy of the SSP muscle. The remaining infraspinatus (ISP) muscle weight increased rapidly after a transient decrease (3 days), which could be induced by posttraumatic immobilization. The IGF‐1 mRNA levels increased transiently at 7 days followed by a gradual increase thereafter in the ISP muscle, and those of IGF‐1 receptor mRNA significantly increased after 3 days. IGF‐1 protein levels biphasically increased (3 and 14 days), then gradually decreased thereafter. The IGF‐1 protein levels tended to show a negative correlation with IGF‐1 mRNA levels. These levels also showed a negative correlation with the ISP muscle weight, indicating that the increase in IGF‐1 secretion may contribute to the ISP muscle growth. The pAkt/Akt protein ratio decreased transiently by 14 days, but recovered later. The IGF‐1 protein levels were negatively correlated with the pAkt/Akt ratio. These results indicate that transection of the SSP tendon activates IGF‐1/Akt signaling in the remaining ISP muscle for structural compensation. Thus, the remaining muscles after RCT can be a target for rehabilitation through the activation of IGF‐1/Akt signaling.

## Abstract (precis)

The objective of this study was to determine if the compensatory hypertrophy of the ISP muscle occurs in our RCT rat models, and if insulin‐like growth factor 1 (IGF‐1)/Akt signaling is activated during compensation. Our hypothesis was that the detachment of the supraspinatus (SSP) tendon may alter the mechanical load in the infraspinatus (ISP) muscle and activate the intracellular signal transduction pathway that may be involved in the compensation.

## Introduction

The shoulder joint lacks inherent bony restraint, and the dynamic stability of the shoulder joint is provided by the rotator cuff musculature, which consists of four muscles: the subscapularis, supraspinatus (SSP), infraspinatus (ISP), and teres minor. These muscles provide dynamic stability to the glenohumeral joint by compressing the humeral head into the glenoid concavity. The balance among the anterior subscapularis, posterior ISP, and teres minor has been proposed to be critical for this function. Several studies have shown that, even when the superior part of the rotator cuff musculature, the SSP, is torn, this anterior‐posterior force balance can provide concavity compression and resist superior displacement of the humeral head despite superior pull of the deltoid muscle during abduction (Thompson et al. 1996; Lee et al. 2000; Parsons et al. 2002).

Among the most common musculoskeletal problems encountered in aging humans, the rotator cuff tear (RCT) (Yamaguchi et al. 2006; Yamamoto et al. 2010) causes disruption of normal shoulder kinematics, pain in motion and loss of muscle strength. Surgical treatment for RCT is sometimes performed to reconstruct the rotator cuff musculature by suturing the torn tendon, and to restore the force balance. Conservative treatment for RCT to train the remaining rotator cuff muscles for functional compensation is also commonly performed. Although rehabilitation training has been recommended for the conservative treatment, a limited availability of information on the mode of compensation of the remaining muscles after RCT made it difficult to design an appropriate rehabilitation program. As an animal model for injury‐induced compensation of skeletal muscle, ablation of the soleus and gastrocnemius muscles in rat has been used (DeVol et al. 1990; Adams and Haddad 1996; Tamaki and Shiraishi 1996). In these studies, it has been reported that the weight of the remaining plantaris muscle was increased significantly.

Insulin‐like growth factor 1 (IGF‐1) and its downstream process, the PI3K/Akt system, play an important role in the growth and differentiation of many organs, including skeletal muscle. Various organs produce IGF‐1, most of which act in a para‐ or autocrine manner. On the other hand, the liver supplies approximately 75% of circulating IGF‐1 (Schwander et al. 1983). However, although selective abolishment of IGF‐1 production in hepatocytes leads to a 75% reduction in circulating IGF‐1 levels, no growth impairment is observed (Matheny et al. 2009). In skeletal muscle, local administration of IGF‐1 induces hypertrophy in rats (Adams and McCue 1998). In addition, skeletal muscle is also able to produce IGF‐1 under several specific conditions such as mechanical loading including exercise. The produced IGF‐1 acts locally in a para‐ or autocrine manner and thus does not alter circulating IGF‐1 levels (DeVol et al. 1990; Bamman et al. 2001; Walker et al. 2004; Petrella et al. 2006). Binding of IGF‐1 to its receptor induces a conformational change in the transphosphorylation of IGF‐1 receptor tyrosine kinase. This interaction induces the subsequent phosphorylation of intracellular downstream factors such as Akt (Glass 2003; Bassel‐Duby and Olson 2006; Philippou et al. 2007; Gundersen 2011).

Akt is a serine/threonine kinase that has emerged as a critical signaling component for the regulation of cellular metabolism, growth, and survival in multiple systems. In skeletal muscle, Akt phosphorylation activates protein synthesis and results in hypertrophy. Conditional activation of Akt in skeletal muscle induces rapid hypertrophy in mice (Lai et al. 2004), and running exercise or nerve stimulation induced Akt phosphorylation in skeletal muscle (Nader and Esser 2001; Sakamoto et al. 2002, 2003).

The objective of this study was to determine if the compensatory hypertrophy of the ISP muscle occurs in our RCT rat models, and if IGF‐1/Akt signaling is activated during compensation. Our hypothesis was that the detachment of the SSP tendon may alter the mechanical load in the ISP muscle and activate the intracellular signal transduction pathway that may be involved in the compensation.

## Material and Methods

### Animals

This study was approved by the Animal Care and Experimentation Committee, Gunma University. All efforts were made to minimize the suffering and number of animals in this study. Thirty‐five male Wistar rats (body weight 240–260 g) used in this study were purchased from SLC Japan, Hamamatsu, Japan. All the rats were housed with food and water ad libitum under controlled temperature (24°C), humidity and illumination (12:12 light–dark cycle; lights on at 6:00 am).

### SSP tendon detachment

**Figure 1.**
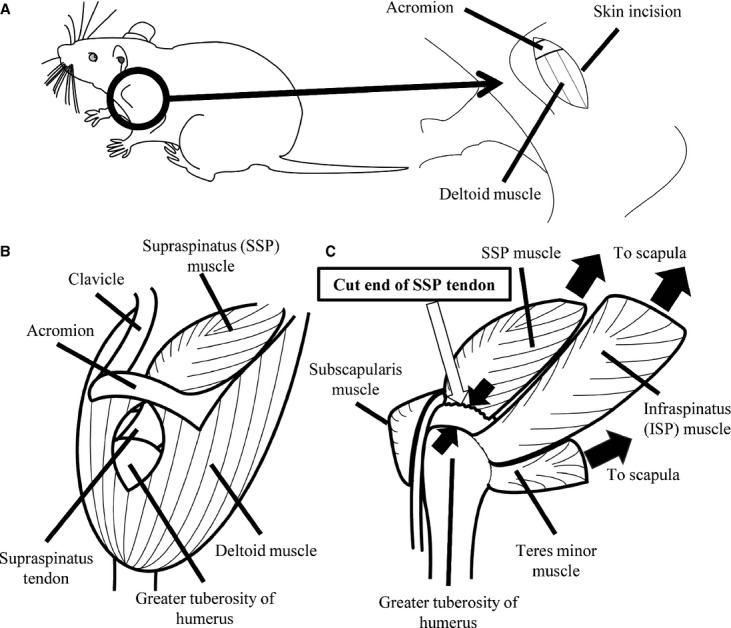
Schematic showing the surgical procedure. Surgical transection of the supraspinatus tendon was performed in the left shoulder. (A) A skin incision was made to expose the rotator cuff tendons. (B) The supraspinatus tendon was exposed by splitting the deltoid muscle (trapezius muscle is not shown in this figure for clarity). (C) The supraspinatus tendon was partly removed from the greater tuberosity of the humerus. For clarity, deltoid muscle, acromion, and clavicle are not shown in this figure.

The rats were divided into seven groups (*n* = 5/group). Under anesthesia with ketamine and xylazine (60 mg of ketamine/kg body weight and 12 mg of xylazine/kg body weight, i.p.), all the rats received surgery for the detachment of the SSP tendon of the left shoulder in accordance with previous reports (Fig. 1) (Barton et al. 2005; Perry et al. 2009; Ward et al. 2010). With the arm in external rotation, a 2 cm skin incision was made, followed by blunt dissection down to the rotator cuff musculature (Fig. 1A). The rotator cuff was exposed, and the tendons were visualized at their insertion on the humerus. The SSP tendon was identified as that passing underneath the acromion. Suture was passed under the acromion to apply upward traction for further exposure, and the SSP tendon was separated from the other rotator cuff tendons before sharp detachment at its insertion on the greater tuberosity using a scalpel blade (Fig. 1B and C). After the detachment, the SSP tendon was partly removed. A 5‐0 nylon suture was used to close the skin, and the rats were allowed unrestricted cage activity.

### Sample collection and preparation

The rats were anesthetized with diethyl ether and sacrificed by decapitation at 0, 3, 7, 14, 28, 56, and 84 days following the tendon detachment. The deltoid muscle was removed carefully after the shoulder girdle was removed. Then the SSP and ISP muscles of left forelimbs were removed from the scapula and humerus. Collected muscles were immediately weighed, frozen in liquid nitrogen and stored at −80°C until use. The ratio of the collected muscle weight to whole body weight was used for subsequent statistical analysis.

### Total RNA isolation and cDNA synthesis

Total RNA from muscle tissue was isolated with Qiagen RNeasy^®^ Lipid Tissue Kit (Qiagen Inc., Valencia, CA) in accordance with the manufacturer's instructions. Briefly, the ISP muscles were homogenized in lysis buffer supplied with the kit. Total RNA was isolated and treated with DNase. The concentration and purity of the isolated RNA were determined by UV spectrophotometry (Beckman Coulter, Inc., Brea, CA). RNA with a 260/280 ratio of 1.5–2.0 was used for cDNA synthesis. cDNA was reverse transcribed from 2.5 *μ*g of total RNA and PrimeScript RT kit (Takara Bio Inc., Otsu, Japan) in a final volume of 50 *μ*L at 37°C for 15 min, followed by 85°C for 5 sec. The synthesized cDNA was dispensed and stored at −80°C until the subsequent polymerase chain reaction.

### Polymerase chain reaction, electrophoresis, and semi‐quantification

cDNA was amplified in 12.5 *μ*L of reaction solution containing appropriate primer pairs and Taq DNA polymerase (Roche Diagnostics GmbH, Mannheim, Germany). Primer sequences and annealing temperatures are shown in Table 1. The thermal cycling program consisted of an initial denaturation step at 95°C for 3 min, followed by an indicated number (Table 1) of cycles at 95°C for 15 sec, a 10‐sec annealing step, and a 45‐sec extension step at 72°C. The optimal number of PCR cycles was determined on the basis of the linearity of amplification. After the last cycle, samples were incubated at 72°C for 5 min, before being held at 4°C. PCR products were diluted 5:1 with loading dye (Ambion, Inc., Austin, TX), and 10 *μ*L of them were analyzed by electrophoresis on a 2.0% agarose gel at 100 V/cm in tris acetate EDTA buffer. The products were visualized with ethidium bromide and quantified with ImageJ Software (NIH). The PCR results for each sample were normalized with glyceraldehyde 3‐phosphate dehydrogenase (GAPDH) mRNA levels as an internal control.

**Table 1.** Primer sequences, annealing temperatures, and amplification cycles for reverse transcription polymerase chain reaction.

Primers	Sequence (5′‐3′)	Length of product (bp)	Annealing temperature	Amplification cycle
GAPDH				
Sense	CAAGGCCGAGAATGGGAAGCT	184	55	20
Antisense	GATGATGACCCTTTTGGCTCC			
IGF‐1				
Sense	AAGCCTACAAAGTCAGCTCG	166	55	37
Antisense	GGTCTTGTTTCCTGCACTTC			
IGF‐1 receptor				
Sense	AAAACCATCGATTCTGTGACG	199	59.5	35
Antisense	GGTTCTTCAGGAAGGACAAGG			

GAPDH, glyceraldehyde 3‐phosphate dehydrogenase.

### Enzyme‐linked immunosorbent assay

The ISP muscles were homogenized with radioimmunoprecipitation assay buffer containing proteinase inhibitors and centrifuged at 10000 *g*. The supernatant was taken and stored at −80°C until use. The total protein concentrations of the samples were determined using a commercially available assay kit (Bio‐Rad Laboratories, Hercules, CA) with bovine serum albumin (BSA) as a standard. After the determination of the total protein concentration, the total IGF‐1 peptide concentration was determined using a commercially available Enzyme‐linked immunosorbent assay (ELISA kit) (R&D Systems, Minneapolis, MN). The IGF‐1 concentration was expressed as pg IGF‐1/mg total protein.

### Western immunoblotting analysis

Protein samples were diluted with Laemmli buffer (Bio‐Rad Laboratories) to 1 *μ*g/*μ*L and heat‐denatured for 4 min at 96°C. Samples (10 *μ*g/lane) were loaded on a 12.5% polyacrylamide gel. The separated proteins were then transferred from the gel to a nitrocellulose membrane (GE Healthcare Inc., Piscataway, NJ) for 1 h at room temperature, and blocked for 1 h at room temperature in 3% blocking reagent (GE Healthcare Inc.) in tris buffered saline (TBS) buffer with 0.1% Tween 20 (TBS‐T). Membranes were incubated overnight at 4°C with rabbit monoclonal anti‐Akt (1:1000) or anti‐phospho‐Akt (pAkt) (ser473) (1:2000) antibodies (both from Cell Signaling Technology Inc, Danvers, MA) in TBS buffer. The subsequent incubation with secondary antibody (anti‐rabbit IgG) conjugated with horseradish peroxidase (1:15000) (GE Healthcare Inc.) was performed at room temperature for 1 h. The signals were developed using enhanced chemiluminescence (ECL) prime reagent (GE Healthcare Inc.) and imaged (Lumi‐Imager F1 LumiImager, Roche Mannheim Boehringer, Mannheim, Germany). The band intensities were determined using ImageJ Software (NIH).

### Statistical analysis

Statistical analysis was performed using SPSS (version 17.0) software (IBM, Chicago, IL). Data were subjected to one‐way analyses of variance (ANOVA) as applicable, following by Tukey's test for post hoc comparison. Correlations between IGF‐1 mRNA levels and the weight ratio of the ISP muscle, IGF‐1 protein, and IGF‐1 mRNA levels, IGF‐1 protein levels and the weight ratio of the ISP muscle, and IGF‐1 protein levels and the ratio of pAkt/Akt were analyzed using Pearson's correlation test. A *P*‐value <0.05 was considered significant in all the analyses.

## Results

### Changes in the SSP and ISP muscle weights

**Figure 2.**
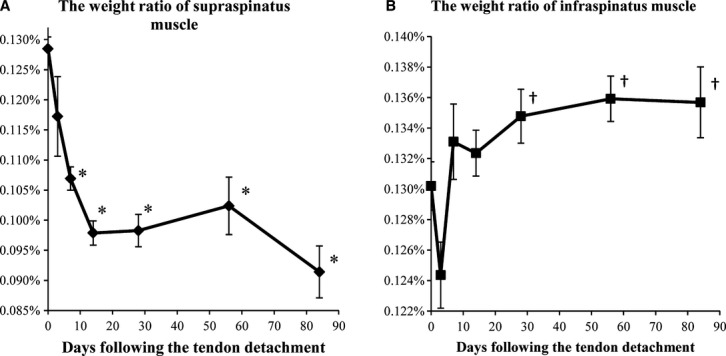
Ratio of the SSP (A) or ISP (B) muscle weight to whole body weight. Data are shown as mean ± SEM. (A) The ratio of the SSP muscle weight significantly decreased at 14 days compared with that at 0 day. This decrease was maintained until 84 days. (B) The weight ratio of the ISP muscle was significantly higher at 28, 56, and 84 days than at 3 days. *significant versus 0 day (*P* < 0.05). ^†^significant versus 3 days (*P* < 0.05).

Normal rats continued to grow during the course of the experiment; for this reason, the muscle weight was normalized to the whole body weight. The ratio of the SSP muscle weight to whole body weight decreased rapidly at 7 days following the tendon detachment, and continued to decrease until 84 days. The ratios were significantly lower at 7, 14, 28, 56, and 84 days than at 0 day, and those were also significantly lower at 14 and 84 days than at 3 days (Fig. 2A). Scar tissue was formed between the end of the SSP tendon and the insertion site of the humerus in all the rats except for the 0 and 3 days group. On the other hand, the ratio of the ISP muscle weight to whole body weight decreased at 3 days, but increased thereafter. The ratios were significantly higher at 28, 56, and 84 days than at 3 days (Fig. 2B). Compared with those at 0 day, the ratios tended to be higher after 7 days (*P* =**0.013, by ANOVA), but no statistical significance by post hoc comparison was observed.

### Changes in IGF‐1 and IGF‐1 receptor mRNA levels

**Figure 3.**
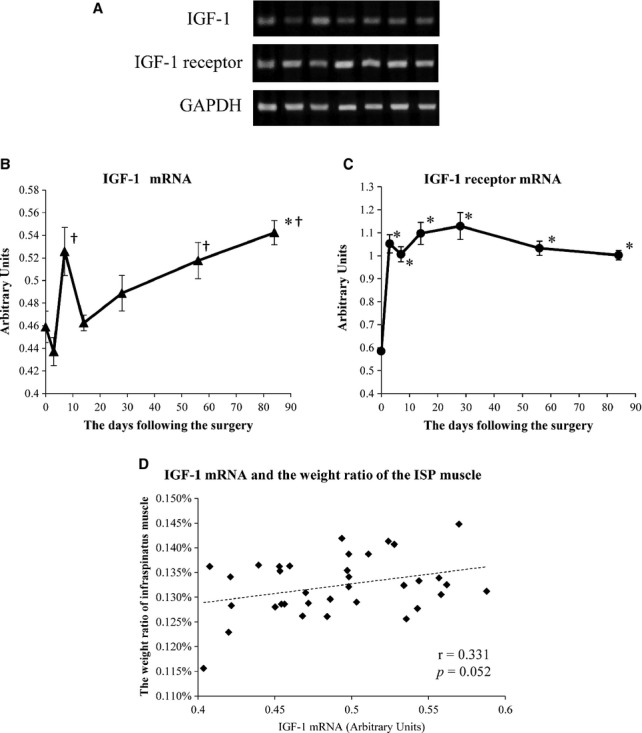
Changes in IGF‐1 and IGF‐1 receptor mRNA levels in the ISP muscle. (A) Representative amplified PCR bands visualized by ethidium bromide staining. (B) Change in relative levels of IGF‐1 mRNA. Data are expressed as arbitrary units, which were normalized with the corresponding density of GAPDH mRNA. The levels were significantly higher at 84 days than at 0 day, and those were also significantly higher at 7, 56, and 84 days than at 3 days. (C) Change in relative levels of IGF‐1 receptor mRNA. IGF‐1 receptor mRNA levels were significantly higher at 3 days following surgery than at 0 day. (D) The IGF‐1 mRNA and the weight ratio of the ISP muscle tended to show a positive correlation, but it was not statistically significant (*r* = 0.331, *P *= 0.052). *significant versus 0 day (*P* < 0.05). ^†^significant versus 3 days (*P* < 0.05).

Representative amplified PCR bands are shown in Fig. 3A. IGF‐1 mRNA levels increased transiently at 7 days (Fig. 3B). Then, it decreased to levels similar to those before the tendon detachment at 14 days. Thereafter, IGF‐1 mRNA levels kept increasing until 84 days, which were significantly higher than those at 0 day, and the levels were also significantly higher at 7, 56, and 84 days than at 3 days (Fig. 3B). IGF‐1 mRNA levels and the ratio of the ISP muscle to the whole body weight tended to show a positive correlation, although it was not statistically significant (*r* = 0.331, *P* =**0.052) (Fig. 3D). IGF‐1 receptor mRNA levels increased significantly at 3 days following the tendon detachment, and remained the same until 84 days (Fig. 3C). IGF‐1 receptor mRNA levels were significantly higher at 3, 7, 14, 28, 56, and 84 days than at 0 day (Fig. 3C).

### Changes in IGF‐1 protein levels

**Figure 4.**
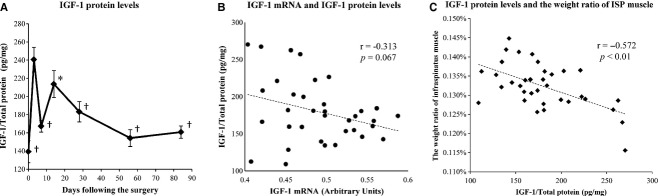
IGF‐1 protein levels measured by ELISA and correlation with IGF‐1 mRNA levels. (A) IGF‐1 protein levels were significantly higher at 3 days than at 0, 7, 28, 56, and 84 days. The levels were also significantly higher at 14 days than at 0 day. (B) The IGF‐1 mRNA and IGF‐1 protein levels tended to show a negative correlation, but it was not statistically significant (*r* = −0.313, *P *= 0.067). (C) The IGF‐1 protein levels and the weight ratio of the ISP muscle were negatively correlation (*r* = −0.572, *P *< 0.01). *significant versus 0 day (*P* < 0.05). ^†^significant versus 3 days (*P* < 0.05).

IGF‐1 protein levels increased transiently at 3 days, decreased significantly at 7 days and increased again at 14 days following the tendon detachment (Fig. 4A). After 14 days, IGF‐1 protein levels decreased gradually. IGF‐1 protein levels were significantly higher at 3 days than at 0, 7, 28, 56, and 84 days. The levels were also significantly higher at 14 days than at 0 day (Fig. 4A). IGF‐1 mRNA and IGF‐1 protein levels tended to show a negative correlation, although it was not statistically significant (*r* = −0.313, *P* =**0.067) (Fig. 4B). Furthermore, IGF‐1 protein levels and the ratio of the ISP muscle weight to whole body weight were negatively correlated (*r* = −0.572, *P *<**0.01) (Fig. 4C).

### Ratio of pAkt to Akt protein levels

**Figure 5.**
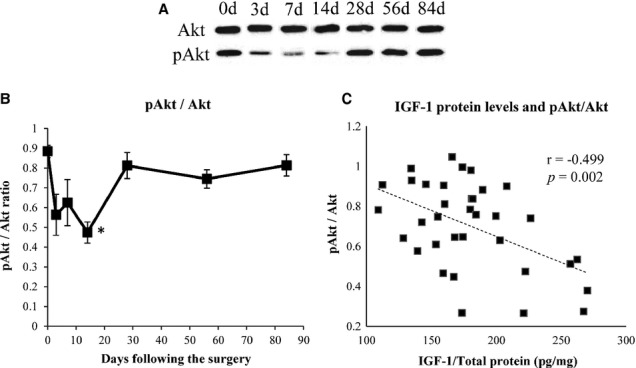
Ratio of pAkt/Akt levels and its correlation with IGF‐1 protein levels. (A) Representative Western blot results of Akt and pAkt. Western blot analysis was carried out using rabbit monoclonal antibodies against Akt or pAkt. (B) Expression ratio of pAkt/Akt in ISP muscle. The ratios at were significantly lower 14 days than at 0 day. (C) IGF‐1 protein levels and pAkt/Akt ratio were negatively correlated (*r* = −0.499, *P *= 0.002). *significant versus 0 day (*P* < 0.05).

Representative Western blot results of Akt and pAkt are shown in Fig. 5A. We used the pAkt/Akt ratio as a phosphorylation scale. The ratio decreased at 3, 7, and 14 days following the tendon detachment, and then increased to the same level as those at 0 day thereafter (Fig. 5B). The ratios were significantly lower at 14 days than at 0 day (Fig. 5B). IGF‐1 protein levels and pAkt/Akt ratio were negatively correlated (*r* = −0.499, *P* = 0.002) (Fig. 5C).

## Discussion

In this study, we have shown that tendon detachment of the SSP muscle decreased its weight (Fig. 2A), whereas the weight of the surrounding ISP muscle increased after a transient decrease (Fig. 2B), which may be induced as a consequence of the loss of movement after the surgery. The IGF‐1 mRNA levels showed an increase in a biphasic pattern (Fig. 3B), whereas IGF‐1 protein levels showed an opposite pattern of changes (Fig. 4A). On the other hand, IGF‐1 receptor mRNA levels became higher at 3 days after the tendon detachment and remained high until 84 days (Fig. 3C). Finally, pAkt/Akt ratios decreased transiently but returned to the basal level with a decrease in IGF‐1 protein level in the ISP muscle. These results indicate that atrophy of the SSP muscle induced a compensatory growth of the surrounding ISP muscle at least in part by the increased secretion of IGF‐1 that may act in a para‐ or autocrine manner.

As shown in Figure 2, the ratio of the SSP muscle weight to whole body weight, which decreased following the tendon detachment, never showed any recovery throughout the observation period. Previous studies have shown that the SSP muscle weight decreased significantly until 6 weeks following the tendon detachment, but recovered at 8 weeks (Barton et al. 2005; Liu et al. 2011). In these reports, however, the muscle weights were compared only on the basis of the wet weight and the effect of general growth was not considered. Even if the tendon is detached, the muscle may keep growing with the increase in general body mass. Thus, as shown in this study, determining the ratio of muscle weight to body weight may be a better method of examining the effect of detachment.

The ratio of the ISP muscle weight to whole body weight decreased temporarily at 3 days following the tendon detachment and then tended to increase thereafter. On the other hand, in rat models that received the soleus and gastrocnemius muscle ablation, the remaining plantaris muscle is well hypertrophied without any temporal decrease in weight (DeVol et al. 1990; Adams and Haddad 1996; Tamaki and Shiraishi 1996). Our RCT model is different from such models under some conditions. One is the degree of increased mechanical load, and the other is the target site, shoulder or hind limb. In previous reports, the increased mechanical load depended on only the plantaris muscle, whereas the increased mechanical load is dispersed to the ISP, teres minor, and subscapularis muscles in our RCT model. Thus, the increase in mechanical load on the ISP muscle may not be as large as that on the plantaris muscle in a previous study. Furthermore, it is not always necessary to use their forelimbs to support their weight when postoperative pain exists. Rats can use their hind limb to move around or to stand up for eating. Thus, a transient disuse of forelimb caused by the postoperative inflammation and pain may have caused a decrease in the ISP weight in the early period following the surgery.

IGF‐1 mRNA levels increased in a biphasic manner following the tendon detachment. Although the exact cause of such biphasic increase was not clarified, at least two distinct factors can be considered. The IGF‐1 mRNA level in the plantaris muscle is also increased in the artificial muscle hypertrophy rat model (DeVol et al. 1990; Adams and Haddad 1996; Adams et al. 1999). In their study, IGF‐1 mRNA levels kept increasing from early to late phase following intervention (exercise and surgery), and they conclude that the IGF‐1 mRNA expression is due to increased mechanical load. In this regard, a gradual increase in IGF‐1 mRNA level with an increase in the ISP muscle weight ratio after 14 days may be induced through a similar mechanism. On the other hand, however, a transient increase in IGF‐1 mRNA level at 7 days may not be due to an increase in mechanical load. Rather, the IGF‐1 mRNA expression may be stimulated by inflammation. Certain inflammatory cytokines, interleukin (IL)‐6 and chemokine (C‐C motif) ligand (CCL) 2, are involved in skeletal muscle hypertrophy or regeneration, and knockout of these cytokine genes inhibits the increase in muscle weight and the IGF‐1 mRNA level in the early period following the intervention (Lu et al. 2011; Washington et al. 2011). Taken together with these previous reports, the present results indicate that postoperative inflammation may mainly induce a transient increase in the IGF‐1 mRNA level in the early period following the tendon detachment, and that the increase in mechanical load mainly stimulates IGF‐1 mRNA expression in the later period, causing a biphasic increase in its expression.

In addition to IGF‐1 mRNA, IGF‐1 receptor mRNA levels also increased significantly from the early period following the tendon detachment and maintained its high level until the end of the observation period. The IGF‐1 receptor is essential for IGF‐1 action. Its transphosphorylation activates subsequent phosphorylation of downstream molecules related to muscle hypertrophy (Philippou et al. 2007). The IGF‐1 receptor mRNA levels in the skeletal muscle increase by resistant or chronic exercise (Haddad and Adams 2002, 2006) and in the artificial hypertrophy rat model (Owino et al. 2001). In addition, chronic exercise not only increases the IGF‐1 receptor mRNA level but also enhances the binding capacity of the IGF‐1 receptor (Willis et al. 1998). From these reports, it seemed that the IGF‐1 receptor expression level in the skeletal muscle is increased to adapt to the increase in mechanical load. Thus, the present results indicate that the expression of the IGF‐1 receptor mRNA in the ISP muscle is stimulated by the increase in mechanical load caused by the SSP tendon detachment.

At 3 days, when IGF‐1 mRNA levels were lower than at 0 day, the IGF‐1 protein levels transiently increased. When the IGF‐1 mRNA level showed a sharp increase at 7 days, on the other hand, its protein level decreased. Then, while the IGF‐1 mRNA level gradually increased from 14 days until the end of the observation period, its protein level tended to decrease. These patterns tended to show a negative correlation. In the skeletal muscle, the IGF‐1 protein is secreted in an autocrine or autocrine/paracrine manner (Glass 2003; Bassel‐Duby and Olson 2006; Philippou et al. 2007; Gundersen 2011), and thus, the intracellular level of IGF‐1 is decreased by secretion into the extracellular space. The present results indicate that IGF‐1 mRNA synthesis is transiently decreased at 3 days, resulting in an increase in the intracellular protein level of IGF‐1 and then, with the increase in IGF‐1 mRNA synthesis, its release was activated, resulting in the decrease in the intracellular protein level of IGF‐1. In the previous study, however, IGF‐1 protein levels in the skeletal muscle increased significantly following the surgical intervention in the artificial hypertrophy rat model in parallel with IGF‐1 mRNA levels (Adams and Haddad 1996). The cause of the difference between this previous study and this study is not known. However, while previous studies showed a clear hypertrophy (50% increases at 6 days and 100% increases at 28 days) of the plantaris muscles following the unilateral or bilateral removal of the gastrocnemius and soleus muscles (Adams and Haddad 1996; Tamaki and Shiraishi 1996), no such strong hypertrophy was observed in this study, indicating that the mechanical load is much lower. Thus, in their study, protein synthesis may have exceeded the speed of secretion, resulting in IGF‐1 protein accumulation in parallel with its mRNA accumulation.

Among biomolecules which were measured in this study, IGF‐1 mRNA levels tended to show a positive correlation with the ratio of the ISP muscle to the whole body weight, and IGF‐1 protein levels were negatively correlated with the ratio of the ISP muscle weight. As mentioned above, increased IGF‐1 mRNA indicates activated synthesis of IGF‐1 protein, and decreased intracellular IGF‐1 protein level indicates activated secretion to the extracellular space. Thus, these correlations suggest that the weight of the ISP muscle was affected by the synthesis and secretion of IGF‐1. The increase in IGF‐1 secretion may at least in part contribute to the ISP muscle growth.

The pAkt/Akt ratios in the ISP muscle decreased until 14 days following the tendon detachment, and increased to the same level as the baseline thereafter. Intracellular IGF‐1 protein levels and pAkt/Akt ratios were negatively correlated, indicating that the Akt pathway may be activated by the secreted IGF‐1. In fact, as shown in Figures 4A and 5B, the changes in IGF‐1 protein levels and pAkt/Akt ratios showed an opposite tendency except those at 7 days. Conditional activation of Akt in the skeletal muscle induces rapid hypertrophy in mice (Lai et al. 2004). Running exercise or nerve stimulation also induced Akt phosphorylation in the skeletal muscle (Nader and Esser 2001; Sakamoto et al. 2002, 2003). Akt is also phosphorylated in the artificial hypertrophy rat model (Bodine et al. 2001). On the other hand, the activation of a certain inflammatory signal molecule, such as JNK, inhibits Akt phosphorylation (Varma et al. 2009). Taken together with these previous reports, the present results indicate that the pAkt/Akt ratios may have first decreased as a result of the combination of postoperative inflammation and decreased IGF‐1 secretion. Then, with an increase in IGF‐1 secretion, Akt signaling may be activated.

As discussed above, we have considered our rat model as a human RCT model. However, there are some limitations in this study for extrapolation. First, although the rat shoulder joint is similar to the human shoulder joint in terms of anatomical structure, the function is slightly different. The rat shoulder partly acts as a weight‐bearing joint, whereas the human shoulder joint is responsible for a part of motor function of the upper extremity as a nonweight‐bearing joint. Second, our RCT model is obtained by surgical procedure, whereas the RCT in human is mainly caused by the degradation of the rotator cuff tendons. Surgical procedure might have led to inflammation, as discussed above, affecting the expression of a variety of biomolecules in the ISP muscle. We may have to establish further the RCT model caused by the degradation of the rotator cuff tendons or to minimize the surgical invasion in making RCT model. Third, the detachment of the SSP tendon could not provide sufficient increase in mechanical load on the remaining ISP muscle to achieve compensatory hypertrophy. We have to create a new RCT model, in which we can observe a definite compensatory hypertrophy on the remaining muscle.

In conclusion, the results of this study showed that the ISP muscle can recover from transient disuse caused by the SSP tendon detachment (tear), and partly compensate for the SSP muscle function. IGF‐1 signaling pathway in the ISP muscle is activated during recovery, indicating the involvement of this signal transduction pathway. Thus, in single‐tendon RCT, the remaining muscles can be a target for rehabilitative training by activating IGF‐1‐mediated signaling.

## Conflict of Interest

None declared.

## References

[b1] Adams G. R. Haddad F. 1996 The relationships among IGF‐1, DNA content, and protein accumulation during skeletal muscle hypertrophy. J. Appl. Physiol.; 81:2509-2516 9018499 10.1152/jappl.1996.81.6.2509

[b2] Adams G. R. McCue S. A. 1998 Localized infusion of IGF‐I results in skeletal muscle hypertrophy in rats. J. Appl. Physiol.; 84:1716-1722 9572822 10.1152/jappl.1998.84.5.1716

[b3] Adams G. R. Haddad F. Baldwin K. M. 1999 Time course of changes in markers of myogenesis in overloaded rat skeletal muscles. J. Appl. Physiol.; 87:1705-1712 10562612 10.1152/jappl.1999.87.5.1705

[b4] Bamman M. M. Shipp J. R. Jiang J. Gower B. A. Hunter G. R. Goodman A. 2001 Mechanical load increases muscle IGF‐I and androgen receptor mRNA concentrations in humans. Am. J. Physiol. Endocrinol. Metab.; 280:E383-E390 11171591 10.1152/ajpendo.2001.280.3.E383

[b5] Barton E. R. Gimbel J. A. Williams G. R. Soslowsky L. J. 2005 Rat supraspinatus muscle atrophy after tendon detachment. J. Orthop. Res.; 23:259-265 15734235 10.1016/j.orthres.2004.08.018

[b6] Bassel‐Duby R. Olson E. N. 2006 Signaling pathways in skeletal muscle remodeling. Annu. Rev. Biochem.; 75:19-37 16756483 10.1146/annurev.biochem.75.103004.142622

[b7] Bodine S. C. Stitt T. N. Gonzalez M. Kline W. O. Stover G. L. Bauerlein R. 2001 Akt/mTOR pathway is a crucial regulator of skeletal muscle hypertrophy and can prevent muscle atrophy in vivo. Nat. Cell Biol.; 3:1014-1019 11715023 10.1038/ncb1101-1014

[b8] DeVol D. L. Rotwein P. Sadow J. L. Novakofski J. Bechtel P. J. 1990 Activation of insulin‐like growth factor gene expression during work‐induced skeletal muscle growth. Am. J. Physiol.; 259:E89-E95 2372054 10.1152/ajpendo.1990.259.1.E89

[b9] Glass D. J. 2003 Signalling pathways that mediate skeletal muscle hypertrophy and atrophy. Nat. Cell Biol.; 5:87-90 12563267 10.1038/ncb0203-87

[b10] Gundersen K. 2011 Excitation‐transcription coupling in skeletal muscle: the molecular pathways of exercise. Biol. Rev. Camb. Philos. Soc.; 86:564-600 21040371 10.1111/j.1469-185X.2010.00161.x PMC3170710

[b11] Haddad F. Adams G. R. 2002 Selected contribution: acute cellular and molecular responses to resistance exercise. J. Appl. Physiol.; 93:394-403 12070230 10.1152/japplphysiol.01153.2001

[b12] Haddad F. Adams G. R. 2006 Aging‐sensitive cellular and molecular mechanisms associated with skeletal muscle hypertrophy. J. Appl. Physiol.; 100:1188-1203 16373446 10.1152/japplphysiol.01227.2005

[b13] Lai K. M. Gonzalez M. Poueymirou W. T. Kline W. O. Na E. Zlotchenko E. 2004 Conditional activation of akt in adult skeletal muscle induces rapid hypertrophy. Mol. Cell. Biol.; 24:9295-9304 15485899 10.1128/MCB.24.21.9295-9304.2004 PMC522257

[b14] Lee S. B. Kim K. J. O'Driscoll S. W. Morrey B. F. An K. N. 2000 Dynamic glenohumeral stability provided by the rotator cuff muscles in the mid‐range and end‐range of motion. A study in cadavera. J. Bone Joint Surg. Am.; 82:849-857 10859105 10.2106/00004623-200006000-00012

[b15] Liu X. Manzano G. Kim H. T. Feeley B. T. 2011 A rat model of massive rotator cuff tears. J. Orthop. Res.; 29:588-595 20949443 10.1002/jor.21266

[b16] Lu H. Huang D. Ransohoff R. M. Zhou L. 2011 Acute skeletal muscle injury: CCL2 expression by both monocytes and injured muscle is required for repair. FASEB J.; 25:3344-3355 21697550 10.1096/fj.10-178939 PMC3177578

[b17] Matheny R. W. Matheny W. Merritt E. Zannikos S. V. Farrar R. P. Adamo M. L. 2009 Serum IGF‐I‐deficiency does not prevent compensatory skeletal muscle hypertrophy in resistance exercise. Exp. Biol. Med. (Maywood); 234:164-170 19064939 10.3181/0808-RM-251

[b18] Nader G. A. Esser K. A. 2001 Intracellular signaling specificity in skeletal muscle in response to different modes of exercise. J. Appl. Physiol.; 90:1936-1942 11299288 10.1152/jappl.2001.90.5.1936

[b19] Owino V. Yang S. Y. Goldspink G. 2001 Age‐related loss of skeletal muscle function and the inability to express the autocrine form of insulin‐like growth factor‐1 (MGF) in response to mechanical overload. FEBS Lett.; 505:259-263 11566187 10.1016/s0014-5793(01)02825-3

[b20] Parsons I. M. Apreleva M. Fu F. H. Woo S. L. 2002 The effect of rotator cuff tears on reaction forces at the glenohumeral joint. J. Orthop. Res.; 20:439-446 12038616 10.1016/S0736-0266(01)00137-1

[b21] Perry S. M. Getz C. L. Soslowsky L. J. 2009 Alterations in function after rotator cuff tears in an animal model. J. Shoulder Elbow Surg.; 18:296-304 19218053 10.1016/j.jse.2008.10.008 PMC2669656

[b22] Petrella J. K. Kim J. S. Cross J. M. Kosek D. J. Bamman M. M. 2006 Efficacy of myonuclear addition may explain differential myofiber growth among resistance‐trained young and older men and women. Am. J. Physiol. Endocrinol. Metab.; 291:E937-E946 16772322 10.1152/ajpendo.00190.2006

[b23] Philippou A. Halapas A. Maridaki M. Koutsilieris M. 2007 Type I insulin‐like growth factor receptor signaling in skeletal muscle regeneration and hypertrophy. J. Musculoskelet. Neuronal Interact.; 7:208-218 17947802

[b24] Sakamoto K. Hirshman M. F. Aschenbach W. G. Goodyear L. J. 2002 Contraction regulation of Akt in rat skeletal muscle. J. Biol. Chem.; 277:11910-11917 11809761 10.1074/jbc.M112410200

[b25] Sakamoto K. Aschenbach W. G. Hirshman M. F. Goodyear L. J. 2003 Akt signaling in skeletal muscle: regulation by exercise and passive stretch. Am. J. Physiol. Endocrinol. Metab.; 285:E1081-E1088 12837666 10.1152/ajpendo.00228.2003

[b26] Schwander J. C. Hauri C. Zapf J. Froesch E. R. 1983 Synthesis and secretion of insulin‐like growth factor and its binding protein by the perfused rat liver: dependence on growth hormone status. Endocrinology; 113:297-305 6190641 10.1210/endo-113-1-297

[b27] Tamaki T. Shiraishi T. 1996 Characteristics of compensatory hypertrophied muscle in the rat: II. Comparison of histochemical and functional properties. Anat. Rec.; 246:335-342 8915455 10.1002/(SICI)1097-0185(199611)246:3<335::AID-AR4>3.0.CO;2-X

[b28] Thompson W. O. Debski R. E. Boardman N. D. Taskiran E. Warner J. J. Fu F. H. 1996 A biomechanical analysis of rotator cuff deficiency in a cadaveric model. Am. J. Sports Med.; 24:286-292 8734877 10.1177/036354659602400307

[b29] Varma V. Yao‐Borengasser A. Rasouli N. Nolen G. T. Phanavanh B. Starks T. 2009 Muscle inflammatory response and insulin resistance: synergistic interaction between macrophages and fatty acids leads to impaired insulin action. Am. J. Physiol. Endocrinol. Metab.; 296:E1300-E1310 19336660 10.1152/ajpendo.90885.2008 PMC2692398

[b30] Walker K. S. Kambadur R. Sharma M. Smith H. K. 2004 Resistance training alters plasma myostatin but not IGF‐1 in healthy men. Med. Sci. Sports Exerc.; 36:787-793 15126711 10.1249/01.mss.0000126384.04778.29

[b31] Ward S. R. Sarver J. J. Eng C. M. Kwan A. Würgler‐Hauri C. C. Perry S. M. 2010 Plasticity of muscle architecture after supraspinatus tears. J. Orthop. Sports Phys. Ther.; 40:729-735 20710096 10.2519/jospt.2010.3279 PMC4321894

[b32] Washington T. A. White J. P. Davis J. M. Wilson L. B. Lowe L. L. Sato S. 2011 Skeletal muscle mass recovery from atrophy in IL‐6 knockout mice. Acta Physiol. (Oxf.); 202:657-669 21418148 10.1111/j.1748-1716.2011.02281.x PMC3129379

[b33] Willis P. E. Chadan S. G. Baracos V. Parkhouse W. S. 1998 Restoration of insulin‐like growth factor I action in skeletal muscle of old mice. Am. J. Physiol.; 275:E525-E530 9725821 10.1152/ajpendo.1998.275.3.E525

[b34] Yamaguchi K. Ditsios K. Middleton W. D. Hildebolt C. F. Galatz L. M. Teefey S. A. 2006 The demographic and morphological features of rotator cuff disease. A comparison of asymptomatic and symptomatic shoulders. J. Bone Joint Surg. Am.; 88:1699-1704 16882890 10.2106/JBJS.E.00835

[b35] Yamamoto A. Takagishi K. Osawa T. Yanagawa T. Nakajima D. Shitara H. 2010 Prevalence and risk factors of a rotator cuff tear in the general population. J. Shoulder Elbow Surg.; 19:116-120 19540777 10.1016/j.jse.2009.04.006

